# Comparative Evaluation of the Prognosis of Septic Shock Patients from Before to After the Onset of the COVID-19 Pandemic: A Retrospective Single-Center Clinical Analysis

**DOI:** 10.3390/jpm12010103

**Published:** 2022-01-13

**Authors:** Jae Hwan Kim, Chiwon Ahn, Myeong Namgung

**Affiliations:** Department of Emergency Medicine, College of Medicine, Chung-Ang University, Seoul 06974, Korea; sult@caumc.or.kr (J.H.K.); myeong15180@caumc.or.kr (M.N.)

**Keywords:** COVID-19, coronavirus, emergency department, sepsis, septic shock, mortality

## Abstract

In this study, we investigated the mortality of septic shock patients visiting emergency departments (ED) before and after the coronavirus disease (COVID-19) pandemic onset. We retrospectively reviewed medical records and National Emergency Department Information System data of septic shock patients who visited the ED of a tertiary medical center in South Korea from February 2019 to February 2021. Following the COVID-19 pandemic onset, revised institutional ED processes included a stringent isolation protocol for patients visiting the ED. The primary goal of this study was to determine the mortality rate of septic shock patients from before and after the onset of the COVID-19 pandemic. Durations of vasopressor use, mechanical ventilation, intensive care unit stay, and hospitalization were investigated. The mortality rates increased from 24.8% to 35.8%, before and after COVID-19-onset, but the difference was not statistically significant (*p* = 0.079). No significant differences in other outcomes were found. Multivariable analysis revealed that the Simplified Acute Physiology Score III (SAPS III) was the only risk factor for mortality (OR 1.07; 95% CI 1.04-1.10), whereas COVID-19 pandemic was not included in the final model. The non-significant influence of the COVID-19 pandemic on septic shock mortality rates in the present study belies the actual mortality-influencing potential of the COVID-19 pandemic.

## 1. Introduction

The coronavirus disease (COVID-19) pandemic that ensued from the novel coronavirus outbreak in December 2019 has induced rapid changes in the healthcare environment [1]. Since the first Korean COVID-19 patient was diagnosed in January 2020, the healthcare system in South Korea has witnessed several crises [2,3]. Due to its rapid spread, COVID-19 has been accorded a higher infectious crisis level, and hospitals have changed their patient acceptance system and isolation protocols to ensure a complete separation of infected patients in order to prevent infection transmission. The majority of hospital resources being allocated toward the diagnosis and treatment of COVID-19 has limited the provision of appropriate treatment to critically ill patients [4,5]. Emergency departments (ED), which provide acute care to patients with emergent conditions, have experienced inadequate medical resource allocation during the COVID-19 pandemic, and this could prove life-threatening for critically ill patients [6,7,8].

Inadequate medical resource distribution and overcrowding of medical facilities may result in facility closure. In a community with concurrent and recurrent closures of a nearby ED during the COVID-19 outbreak, the hospital mortality rate, intensive care unit (ICU) admission rate, and length of ED stay for patients who needed admission increased [9]. Furthermore, the trends in the usage of medical services and clinical outcomes of patients with acute and chronic diseases, such as cardiovascular disease, have been affected by the COVID-19 pandemic [10,11]. Septic shock is a critical illness with a high mortality rate and, therefore, early diagnosis and initiation of antibiotic therapy after symptom onset are crucial for improving patient outcomes [12]. With this is in mind, COVID-19 cannot be ruled out merely based on a clinical examination of patients with septic shock who visit the ED, and, furthermore, delaying access to care, as part of infection-control protocols to prevent the spread of COVID-19, will inevitably hinder the provision of timely treatment. 

To our knowledge, no study has investigated whether COVID-19 has affected the clinical outcome of patients with septic shock. Therefore, this study was conducted with an aim to ascertain the differences in mortality rates and clinical outcomes of patients with septic shock who visited the ED before and after the onset of the COVID-19 pandemic.

## 2. Materials and Methods

### 2.1. Study Design and Population

This retrospective study was performed by reviewing data, obtained from the electronic medical records (EMR) and the National Emergency Department Information System (NEDIS), of patients with septic shock who visited a single tertiary emergency medical center in the Republic of Korea from February 2019 to February 2021. The NEDIS is a nationwide emergency-care information network operated by the Ministry of Health and Welfare, and this database includes the demographics and clinical data of all patients who have visit EDs in South Korea. The study center has a 30-bed ED staffed by board-certified emergency physicians who treat approximately 44,000 patients per year while providing 24 h emergency service.

We included all adult septic shock patients (age ≥ 18 years) who were admitted through the ED. Two investigators reviewed the EMR and NEDIS to identify patients with a diagnosis of septic shock in accordance with the International Classification of Diseases 10th Revision (ICD–10) code R572. Septic shock was defined as a clinical illness that fulfills the criteria for sepsis and, despite adequate fluid resuscitation, necessitates vasopressor use to maintain a mean arterial pressure (MAP) ≥ 65 mmHg and serum lactate level > 2 mmol/L (>18 mg/dL). We excluded patients who: (1) had been misdiagnosed; (2) were transferred to other hospitals before admission; (3) had missing records; (4) were revisiting; and (5) were younger than 18 years.

**Figure 1:**
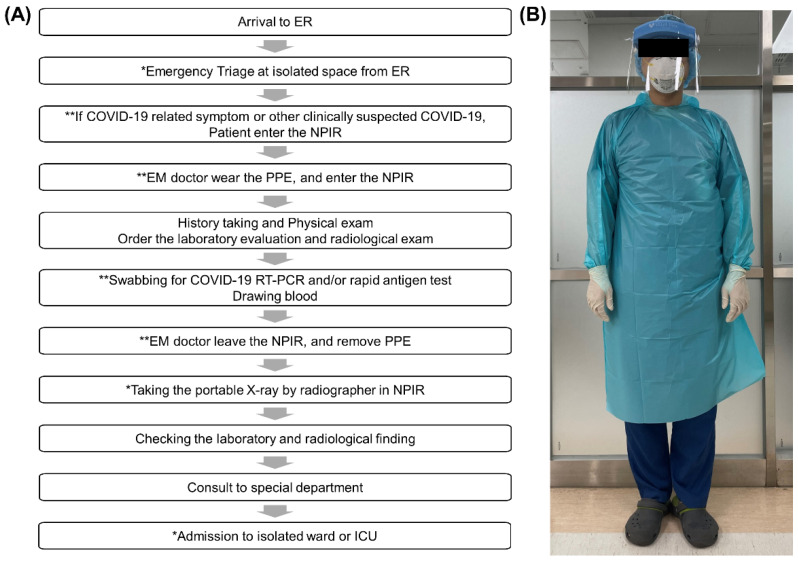
Clinical algorithm for patients visiting the ED, and a picture of an ED worker wearing PPE after the COVID-19 pandemic. A star (*) indicates changes in the process, and a double star (**) indicates an addition in the process with regard to the previous clinical pathway. (**A**) Clinical pathway. (**B**) ED worker wearing PPE. Acronyms: ED, emergency department; COVID-19, coronavirus disease; EM, emergency medicine; NPIR, negative pressure isolation room; RT-PCR, reverse transcription-polymerase chain reaction; ICU, intensive care unit; ER; emergency room; and PPE; personal protective equipment.

In accordance with the upgradation of the infectious disease crisis level to “serious” by the Korean government in February 2020, the emergency medical staff and the Infection Control Team established and modified the ED clinical processes in order to prevent infection spread in the ED during the COVID-19 pandemic on 10 February 2020 [13]. We used this date to classify patients into pre-COVID-19 and post-COVID-19-onset groups (each for a 1-year duration). According to the modified clinical algorithm (Figure 1A), which was implemented due to the COVID-19 pandemic, the triage space was set up in a separate space outside the ED building. Following screening, if patients had COVID-19-related symptoms or were clinically suspected of COVID-19, they entered the negative pressure isolation room (NPIR) instead of the space for patients with other general symptoms. Emergency room (ER) workers, including staff, residents, nurses, and healthcare assistants, wore personal protective equipment (PPE) that consisted of a protective gown, a protective cap, gloves, an N95 mask, and a face shield or goggles (Figure 1B) [14]. All patients staying in the isolated room underwent a COVID-19 reverse transcription-polymerase chain reaction test. If the decision to hospitalize was determined before the result of a confirmatory COVID-19 test, the patients were moved to the isolation ICU or isolation wards through a separate path.

### 2.2. Data Collection and Outcome Measurement

Demographics included sex, age, mode of ED arrival, time from onset of initial symptoms to ED arrival, and comorbidities. Data were recorded on factors associated with the treatment of septic shock patients in the ED, such as the quick Sequential Organ Failure Assessment (qSOFA) score, the Simplified Acute Physiology Score III (SAPS III), infection site, admission type, duration of ED stay (days), time from ED arrival to blood culture, time from ED arrival to administration of antibiotics, time from ED arrival to computed tomography (CT), and time from ED arrival to emergency intervention. 

The primary goal of this study was to determine the in-hospital mortality rate of septic shock patients from before and after the onset of the COVID-19 pandemic. As secondary outcomes, we compared the duration (in days) of vasopressor use, mechanical ventilation, continuous renal replacement therapy (CRRT), ICU stay, and total hospitalization from before and after the onset of the COVID-19 pandemic.

### 2.3. Statistical Analysis

We used SPSS for Windows (version 26, IBM Corporation, Armonk, NY, USA) and R program (version 4.1.1, The R Foundation for Statistical Computing, Vienna, Austria) for all statistical analyses. Descriptive statistical analysis was applied to ascertain baseline characteristics. For continuous variables, values are presented as the mean ± standard deviation. Intergroup differences in normally distributed variables were analyzed using the Student’s *t*-test. Categorical variables are expressed as frequencies and percentages. The chi-square or Fisher’s exact test was used to analyze categorical variables using contingency tables.

To identify predictors of outcomes, the intergroup covariates, including the binary variable of COVID-19 (before or after the onset of the COVID-19 pandemic), were evaluated by multivariate analysis, which was performed independently by logistic regression using the “enter” method. Sex, age, time from onset of initial symptom to ED arrival, time from arrival to administration of antibiotics, SAPS III, and COVID-19 pandemic were adjusted. A *p*-value < 0.05 was considered statistically significant.

### 2.4. Ethics Statement

This study protocol was approved by the Institutional Review Board of the Chung-Ang University Hospital in November 2021 (IRB No. 2109-030-19387). The requirement for informed consent was waived owing to the retrospective nature of the study.

## 3. Results

**Figure 2:**
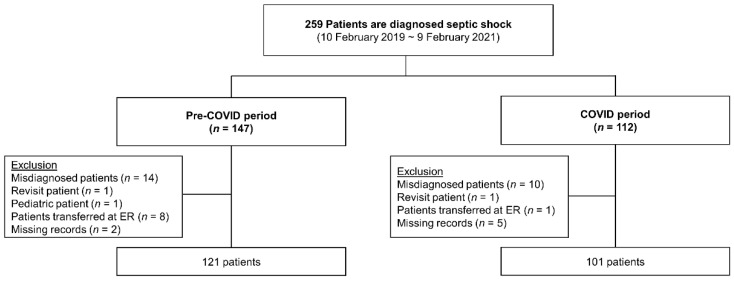
Flow chart of patients enrolled from the NEDIS. Acronyms: NEDIS, National Emergency Department Information System; COVID-19, coronavirus disease; and ER, emergency room.

A total of 259 patients were classified as septic shock. In the pre-COVID-19 period, 147 patients were screened, and, from these, 26 were excluded due to misdiagnosis, revisit, age < 18 years, transfer to other facilities, and insufficient medical record; the final analysis dataset included 121 patients in the pre-COVID-19 group. In the post-COVID-19-onset period, 112 patients were identified, of whom 17 were excluded due to misdiagnosis, revisit, transfer to other facilities, and insufficient medical record; 95 patients were included in the final analysis dataset of the post-COVID-19–onset group (Figure 2). 

### 3.1. Baseline Characteristics

In this study cohort, the mean age was 75.4 ± 12.9 years, and 56.5% of patients were transferred to the ED from other institutions. In the pre- and post–COVID-19–onset periods, the mean values of time from the onset of initial symptoms to ED arrival were 8.7 (2.9–18.2) and 10.6 (2.3–68.8) hours (*p* = 0.090). Furthermore, the qSOFA and SAPS III were 1.4 ± 0.8 and 1.4 ± 0.9 (*p* = 0.849), and 63.8 ± 15.5 and 67.1 ± 15.0 (*p* = 0.114), respectively. There was no intergroup difference in comorbidities, except for hypertension and chronic renal disease; a higher proportion of patients in the pre–COVID-19 group had hypertension than those in the post-COVID-19-onset group (54.5% vs. 41.4%; *p* = 0.049; Table 1). None of the study participants tested positive for the severe acute respiratory syndrome coronavirus 2 infection on RT-PCR testing.

### 3.2. Clinical Characteristics

There was no intergroup difference in: the time from ED arrival to blood culture; the time from ED arrival to administration of antibiotics; the time from ED arrival to CT; the time from ED arrival to emergency intervention; and the infection sites between the pre-COVID-19 and post–COVID-19-onset groups. Statistical analysis of CT and emergency interventions only included patients who underwent the investigation/intervention.

After the onset of COVID-19, the ED stay increased significantly compared to before the COVID-19 pandemic (5.9 ± 3.0 h vs. 5.0 ± 2.3 h; *p* = 0.015). In contrast, ICU admissions decreased after COVID-19 onset in comparison to those in the pre-COVID-19 period (79.3% vs. 66.3%; *p* = 0.023). One patient, who died in the ED while awaiting ICU admission, was excluded from the statistical analysis (Table 1).

### 3.3. Outcomes

The mortality rates from before and after the onset of the COVID-19 pandemic were 24.8% and 35.8%, respectively, and the intergroup difference was not statistically significant (*p* = 0.079). There was no significant intergroup difference in the duration of vasopressor use (3.3 ± 3.3 days vs. 4.3 ± 4.8 days; *p* = 0.057), the application of mechanical ventilation (31.4% vs. 23.2%; *p* = 0.179), the duration of mechanical ventilation (7.8 ± 9.8 days vs. 7.2 ± 7.1 days; *p* = 0.798), the application of CRRT (17.4% vs. 17.9%; *p* = 0.918), the duration of CRRT (3.7 ± 3.7 days vs. 7.7 ± 9.8 days; *p* = 0.129), the ICU stay (4.0 (3.0–9.0) days vs. 5.0 (3.0–8.0) days; *p* = 0.667), and the total hospitalization period (15.0 (8.0–26.0) days vs. 15.0 (7.0–28.0) days; *p* = 0.831) (Table 2).

### 3.4. Multivariable Logistic Regression to Identify Independent Risk Factors of Mortality

**Figure 3:**
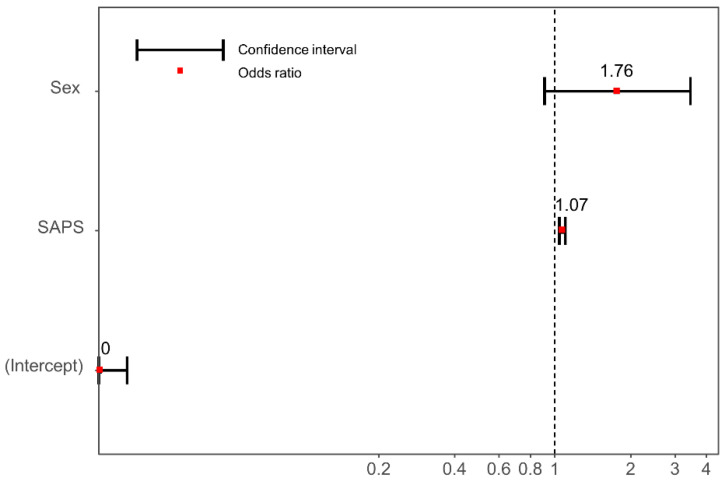
Independent predictors of in-hospital mortality for septic shock patients. Sex, age, time from onset of initial symptoms to ED arrival, time from arrival to administration of antibiotics, the SAPS III, and the pre-COVID-19 and post-COVID-19-onset periods were adjusted. The final model included the following potential risk factors: sex and the SAPS III. Error bars indicate 95% confidence interval and red dots indicates odds ratio. Acronyms: COVID-19, coronavirus disease; and SAPS, Simplified Acute Physiology Score III.

Multivariate logistic regression analysis revealed that the SAPS III was the only independent risk factor for mortality in patients with septic shock (OR 1.07; 95% CI 1.04–1.10; *p* < 0.001); however, the COVID-19 pandemic, as a factor, was not included in the final regression model (Figure 3).

## 4. Discussion

This single-institution retrospective study investigated mortality-related factors in septic shock patients in the pre-COVID-19 and post-COVID-19 onset periods. The results revealed a statistically non-significant influence of the COVID-19 pandemic on the mortality rates of septic shock patients. As compared to the pre-COVID-19 period, there was an apparent but statistically non-significant difference in the time to ER visit from the onset of initial symptoms. The SAPS III, which reflects disease severity, was the only significant independent mortality-associated risk factor. 

In the COVID-19 pandemic era, various studies on COVID-19 are being conducted, and the real-world clinical environments and healthcare systems are more focused on the diagnosis and treatment of COVID-19 patients [4,5,15,16]. We inferred that this paradigm shift would interrupt and delay the provision of appropriate treatment for other critical illnesses, including septic shock; therefore, we investigated the mortality rates of patients with septic shock from before and after the onset of the COVID-19 pandemic. 

The ED constitutes a perennially crowded clinical department where patients with emergency symptoms of various diseases visit. In particular, the overcrowding of EDs during epidemics can increase the possibility of cross-transmission of infectious diseases and contamination of EDs, and this constitutes a potential inception of mass infection [17,18]. The rapid spread of the Middle East Respiratory Syndrome coronavirus in South Korea was attributed to the viral spread in an unspecified number of ED patients [17]. Therefore, in a special situation, such as a pandemic, it is inevitable that the issue of complete isolation of infected patients in the ED is prioritized over the provision of severity-based timely treatment. Furthermore, our institution established a stringent isolation protocol for patients visiting the ED compared to the protocols that were enforced before the COVID-19 pandemic. In cases with suspected or symptomatic COVID-19, patients and medical staff will undertake additional processes before their first encounter with the patient, and clinical management is undertaken in an isolation room, separated from the main ED treatment area. The additional process incurs a time-effort cost for each of the medical personnel to don PPE and enter the isolation room in addition to the time-effort for the allocation of a limited number of isolation rooms.

Furthermore, situations wherein ED medical resources are focused on the diagnosis and treatment of COVID-19 may delay hospital visits for emergency patients with suspected severe infections, including septic shock. In addition, the lack of isolation rooms and shortage of skilled manpower in the local area makes it difficult for patients to visit the hospital. In fact, previous studies and reports have evaluated or anticipated the abovementioned problems [19,20,21]. In this study, before the COVID-19 pandemic, it took 29.5 ± 75 h from illness onset to hospital arrival, whereas the duration extended to 67.1 ± 203.2 h after the onset of the COVID-19 pandemic. However, as this study did not undertake a detailed systematic investigation of the pre–hospitalization situation or the availability of isolation rooms and manpower in hospitals, further research may be necessary to definitively confirm this difference.

The increase of ED stay time was affected by the COVID-19 pandemic. Bae et al. confirmed that the overall length of ED stays of fever patients increased from before to after the onset of the COVID-19 pandemic [22], and the ED stay duration of septic shock patients increased significantly after the onset of the COVID-19 pandemic (pre-COVID-19 pandemic: 5.0 ± 2.3 h vs. post-onset of the COVID-19 pandemic: 5.9 ± 3.0 h; *p* = 0.015). These authors also concluded that the time to antibiotic administration significantly increased for pyrexic patients who visited the ED; however, in the present study, the time to antibiotic administration did not increase in severely ill patients with septic shock (2.4 ± 1.6 h vs. 2.2 ± 1.3 h; *p* = 0.288) [22]. In addition, the present study did not investigate the effects of delayed CT exam or interventions.

The study’s outcomes, including the duration of vasopressor use, mechanical ventilation, ICU stay, and total hospitalization did not change significantly from before to after the onset of the COVID-19 pandemic. Although COVID-19 was included as a mortality-associated variable in the logistic regression analysis, a direct comparison between the two groups did not show statistically significant results in mortality. Contrary to the initial hypothesis, we confirmed that a treatment environment that was focused on COVID-19 patients during the COVID-19 pandemic did not have a direct effect on the mortality of septic shock patients.

This study has several limitations. First, as this is a single-institution study, the overall sample size is small, the interpretation of the results is limited to patients in similar institutions, and, despite investigating the prognosis of severely ill patients after the onset of the COVID-19 pandemic, the results are not widely generalizable. ED visits may vary by hospital size and region, and the spread of COVID-19 may also vary by region. Therefore, it is necessary to conduct additional research by collecting data from various institutions in a multicenter study. Second, investigations of changes in some ED environments were insufficient. Especially, in this study, we did not investigate the shortage of skilled manpower in hospitals or institutions that provided patient care both before and after the onset of COVID-19. The lack of skilled manpower has been previously identified as an important factor that could affect the patient’s treatment outcome [19,20,21]. Further research on this aspect is needed in future studies. Third, we selected patients with septic shock using the main diagnosis code of the NEDIS and selected final dataset of subjects through chart review. However, if the main diagnosis was incorrectly entered in the NEDIS, patients may have been excluded from the study even if they had septic shock. In addition, as this was a retrospective study, information on long-term prognosis could not be obtained. Fourth, in the present study, with the exception of the SAPS III, we did not utilize other severity assessment scoring systems for evaluating the disease severity. Although the mortality risk of patients with septic shock may increase based on disease severity [23], despite the availability of various severity assessment tools such as the Sequential Organ Failure Assessment (SOFA) and the Acute Physiology and Chronic Health Evaluation II (APACHE II), other assessment scores were not evaluated in this study. Fifth, the ED treatment process may differ depending on the cause of septic shock; however, this aspect has not been evaluated in the present study. In addition, mortality rates may differ depending on the cause of septic shock [24], but this factor was not considered in our analysis. 

## 5. Conclusions

The COVID-19 pandemic did not appear to be a factor strongly influencing the mortality rates of patients with septic shock. In addition, there was no significant difference in the use and duration of mechanical ventilation and CRRT in septic shock patients from before to after the onset of the COVID-19 pandemic. The disease severity significantly affected the mortality rate. Nonetheless, we infer that the COVID-19 pandemic has the potential to affect mortality rates in patients with septic shock because of the delay in the ED visits of critically ill patients as well as the increased duration of ED stays.

## Figures and Tables

**Table 1:** Comparison of baseline and clinical characteristics in the pre-COVID-19 and post-COVID-19-onset groups.

Characteristics	Pre-COVID-19 (*N* = 121)	Post-COVID-19-Onset (*N* = 95)	Total (*N* = 216)	*p*-Value
Sex, Male	62 (51.2%)	46 (48.4%)	108 (50.0%)	0.681
Age (years)	75. 4± 12.7	75.4 ± 13.1	75.4 ± 12.9	0.979
Form of ED arrival				
Direct visit Transfer-in	48 (39.7%) 73 (60.3%)	46 (48.4%) 49 (51.6%)	94 (43.5%) 122 (56.5%)	0.198
Time from onset to ED arrival (h)	8.7 (2.9–18.2)	10.6 (2.3–68.8)	9.4 (2.7–35.7)	0.090
qSOFA score	1.4 ± 0.8	1.4 ± 0.9	1.4 ± 0.8	0.849
SAPS III	63.8 ± 15.5	67.1 ± 15.0	65.2 ± 15.3	0.114
Comorbidities				
Hypertension	66 (54.5%)	39 (41.1%)	105 (48.6%)	0.049
Diabetes	38 (31.4%)	36 (37.9%)	74 (34.3%)	0.318
Cerebrovascular disease	25 (20.7%)	17 (17.9%)	42 (19.4%)	0.610
Malignancy	22 (18.2%)	21 (22.1%)	43 (19.9%)	0.474
Cardiovascular disease	14 (11.6%)	12 (12.6%)	26 (12.0%)	0.812
Chronic liver disease	7 (5.8%)	7 (7.4%)	14 (6.5%)	0.639
Chronic renal disease	6 (5.0%)	13 (13.7%)	19 (8.8%)	0.025
Chronic lung disease	4 (3.3%)	6 (6.3%)	10 (4.6%)	0.296
Time from ED arrival to blood culture (h)	1.1 ± 0.9	1.1 ± 0.9		0.592
Time from ED arrival to administration of antibiotics (h)	2.4 ± 1.6	2.2 ± 1.3		0.288
Time from ED arrival to CT (h) (*n* = 158)	2.9 ± 1.0 (*n =* 90)	3.2 ± 1.6 (*n =* 68)		0.136
Time from ED arrival to emergency intervention (h) (*n =* 18)	5.6 ± 3.9 (*n =* 10)	3.9 ± 1.1 (*n =* 8)		0.267
Infection site				
Respiratory	47 (38.8%)	25 (26.3%)	72 (33.3%)	0.053
Urinary	45 (37.2%)	47 (49.5%)	92 (42.6%)	0.070
Hepatobiliary	16 (13.2%)	15 (15.8%)	31 (14.4%)	0.593
Gastrointestinal	9 (7.4%)	10 (10.5%)	19 (8.8%)	0.426
Bone or soft tissue	6 (5.0%)	3 (3.2%)	9 (4.2%)	0.511
Other or unknown	8 (6.6%)	6 (6.3%)	14 (6.5%)	0.930
Duration of ED stay (h)	5.0 ± 2.3	5.9 ± 3.0		0.015
Admission type (*n =* 215) ^1^				
ICU Ward	96 (79.3%) 24 (20.7%)	63 (66.3%) 32 (33.7%)	159 (73.6%) 56 (36.4%)	0.023

Values are shown as the mean ± standard deviation, median (interquartile range), or frequency (proportion). Acronyms: COVID-19, coronavirus disease; qSOFA, quick Sequential Organ Failure Assessment; SAPS-III, Simplified Acute Physiology Score III; ED, emergency department; B/C, blood culture; CT, computed tomography; and ICU, intensive care unit. ^1^ One patient died in the ED.

**Table 2:** Comparison of outcomes between the pre-COVID-19 and post-COVID-19-onset groups.

Characteristics	Pre–COVID-19 (*N =* 121)	Post-COVID-19-Onset (*N =* 95)	*p*-Value
Mortality	30 (24.8%)	34 (35.8%)	0.079
Duration of vasopressor use (days)	3.3 ± 3.3	4.3 ± 4.8	0.057
Application of mechanical ventilation	38 (31.4%)	22 (23.2%)	0.179
Duration of mechanical ventilation (days)	7.8 ± 9.8	7.2 ± 7.1	0.798
Application of CRRT	21 (17.4%)	17 (17.9%)	0.918
Duration of CRRT (days)	3.7 ± 3.7	7.7 ± 9.8	0.129
Duration of ICU stay (days) (*n =* 159)	4.0 (3.0–9.0) (*n =* 96)	5.0 (3.0–8.0) (*n =* 63)	0.667
Duration of total hospitalization (days)	15.0 (8.0–26.0)	15.0 (7.0–28.0)	0.831

Values are shown as the mean ± standard deviation, median (interquartile range), or frequency (proportion). Acronyms: COVID-19, coronavirus disease; CRRT, continuous renal replacement therapy; and ICU, intensive care unit.

## Data Availability

The datasets generated during the current study are available from the corresponding author on reasonable request.
